# Search for a 'Tree of Life' in the thicket of the phylogenetic forest

**DOI:** 10.1186/jbiol159

**Published:** 2009-07-13

**Authors:** Pere Puigbò, Yuri I Wolf, Eugene V Koonin

**Affiliations:** 1 National Center for Biotechnology Information, National Library of Medicine, National Institutes of Health, Bethesda, MD 20894, USA

## Abstract

**Background:**

Comparative genomics has revealed extensive horizontal gene transfer among prokaryotes, a development that is often considered to undermine the 'tree of life' concept. However, the possibility remains that a statistical central trend still exists in the phylogenetic 'forest of life'.

**Results:**

A comprehensive comparative analysis of a 'forest' of 6,901 phylogenetic trees for prokaryotic genes revealed a consistent phylogenetic signal, particularly among 102 nearly universal trees, despite high levels of topological inconsistency, probably due to horizontal gene transfer. Horizontal transfers seemed to be distributed randomly and did not obscure the central trend. The nearly universal trees were topologically similar to numerous other trees. Thus, the nearly universal trees might reflect a significant central tendency, although they cannot represent the forest completely. However, topological consistency was seen mostly at shallow tree depths and abruptly dropped at the level of the radiation of archaeal and bacterial phyla, suggesting that early phases of evolution could be non-tree-like (Biological Big Bang). Simulations of evolution under compressed cladogenesis or Biological Big Bang yielded a better fit to the observed dependence between tree inconsistency and phylogenetic depth for the compressed cladogenesis model.

**Conclusions:**

Horizontal gene transfer is pervasive among prokaryotes: very few gene trees are fully consistent, making the original tree of life concept obsolete. A central trend that most probably represents vertical inheritance is discernible throughout the evolution of archaea and bacteria, although compressed cladogenesis complicates unambiguous resolution of the relationships between the major archaeal and bacterial clades.

## Background

The tree of life is, probably, the single dominating metaphor that permeates the discourse of evolutionary biology, from the famous single illustration in Darwin's On the *Origin of Species *[1] to 21st-century textbooks. For about a century, from the publication of the *Origin *to the founding work in molecular evolution carried out by Zuckerkandl and Pauling in the early 1960s [2,3], phylogenetic trees were constructed on the basis of phenotypic differences between organisms. Accordingly, every tree constructed during that century was an 'organismal' or 'species' tree by definition; that is, it was assumed to reflect the evolutionary history of the corresponding species. Zuckerkandl and Pauling introduced molecular phylogeny, but for the next two decades or so it was viewed simply as another, perhaps most powerful, approach to the construction of species trees and, ultimately, the tree of life that would embody the evolutionary relationships between all lineages of cellular life forms. The introduction of rRNA as the molecule of choice for the reconstruction of the phylogeny of prokaryotes by Woese and co-workers [4,5], which was accompanied by the discovery of a new domain of life – the Archaea – boosted hopes that the detailed, definitive topology of the tree of life could be within sight.

Even before the advent of extensive genomic sequencing, it had become clear that biologically important common genes of prokaryotes had experienced multiple horizontal gene transfers (HGTs), so the idea of a 'net of life' potentially replacing the tree of life was introduced [6,7]. Advances in comparative genomics revealed that different genes very often had distinct tree topologies and, accordingly, that HGT seemed to be extremely common among prokaryotes (bacteria and archaea) [8-17], and could also have been important in the evolution of eukaryotes, especially as a consequence of endosymbiotic events [18-21]. These findings indicate that a true, perfect tree of life does not exist because HGT prevents any single gene tree from being an accurate representation of the evolution of entire genomes. The nearly universal realization that HGT among prokaryotes is common and extensive, rather than rare and inconsequential, led to the idea of 'uprooting' the tree of life, a development that is often viewed as a paradigm shift in evolutionary biology [11,22,23].

Of course, no amount of inconsistency between gene phylogenies caused by HGT or other processes can alter the fact that all cellular life forms are linked by a tree of cell divisions (*Omnis cellula e cellula*, quoting the famous motto of Rudolf Virchow – paradoxically, an anti-evolutionist [24]) that goes back to the earliest stages of evolution and is only violated by endosymbiotic events that were key to the evolution of eukaryotes but not prokaryotes [25]. Thus, the travails of the tree of life concept in the era of comparative genomics concern the tree as it can be derived by the phylogenetic (phylogenomic) analysis of genes and genomes. The claim that HGT uproots the tree of life more accurately has to be read to mean that extensive HGT has the potential to result in the complete decoupling of molecular phylogenies from the actual tree of cells. It should be kept in mind that the evolutionary history of genes also describes the evolution of the encoded molecular functions, so the phylogenomic analyses have clear biological connotations. In this article we discuss the phylogenomic tree of life with this implicit understanding.

The views of evolutionary biologists on the changing status of the tree of life (see [23] for a conceptual discussion) span the entire range from persistent denial of the major importance of HGT for evolutionary biology [26,27]; to 'moderate' overhaul of the tree of life concept [28-33]; to radical uprooting whereby the representation of the evolution of organisms (or genomes) as a tree of life is declared meaningless [34-36]. The moderate approach maintains that all the differences between individual gene trees notwithstanding, the tree of life concept still makes sense as a representation of a central trend (consensus) that, at least in principle, could be elucidated by comprehensive comparison of tree topologies. The radical view counters that the reality of massive HGT renders illusory the very distinction between the vertical and horizontal transmission of genetic information, so that the tree of life concept should be abandoned altogether in favor of a (broadly defined) network representation of evolution [17]. Perhaps the tree of life conundrum is epitomized in the recent debate on the tree that was generated from a concatenation of alignments of 31 highly conserved proteins and touted as an automatically constructed, highly resolved tree of life [37], only to be dismissed with the label of a 'tree of one percent' (of the genes in any given genome) [38].

Here we report an exhaustive comparison of approximately 7,000 phylogenetic trees for individual genes that collectively comprise the 'forest of life' and show that this set of trees does gravitate to a single tree topology, but that the deep splits in this topology cannot be unambiguously resolved, probably due to both extensive HGT and methodological problems of tree reconstruction. Nevertheless, computer simulations indicate that the observed pattern of evolution of archaea and bacteria better corresponds to a compressed cladogenesis model [39,40] than to a 'Big Bang' model that includes non-tree-like phases of evolution [36]. Together, these findings seem to be compatible with the 'tree of life as a central trend' concept.

## Results and discussion

### The forest of life: finding paths in the thicket

**Figure 1:**
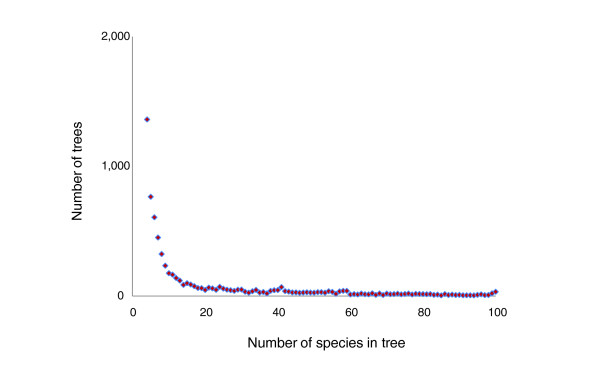
The distribution of the trees in the forest of life by the number of species.

Altogether, we analyzed 6,901 maximum likelihood phylogenetic trees that were built for clusters of orthologous groups of proteins (COGs) from the COG [41,42] and EggNOG [43] databases that included a selected, representative set of 100 prokaryotes (41 archaea and 59 bacteria; Additional data files 1 and 2). The majority of these trees include only a small number of species (less than 20): the distribution of the number of species in trees shows an exponential decay, with only 2,040 trees including more than 20 species (Figure 1). We attempted to identify patterns in this collection of trees (forest of life) and, in particular, to address the question whether or not there exists a central trend among the trees that, perhaps, could be considered an approximation of a tree of life. The principal object of this analysis was a complete, all-against-all matrix of the topological distances between the trees (see Materials and methods for details). This matrix was represented as a network of trees and was also subject to classical multidimensional scaling (CMDS) analysis aimed at the detection of distinct clusters of trees. We further introduced the inconsistency score (IS), a measure of how representative the topology of the given tree is of the entire forest of life (the IS is the fraction of the times the splits from a given tree are found in all trees of the forest). The key aspect of the tree analysis using the IS is that we objectively examine trends in the forest of life, without relying on the topology of a preselected 'species tree' such as a supertree used in the most comprehensive previous study of HGT [31] or a tree of concatenated highly conserved proteins or rRNAs [17,37,44].

In general, trees consist of different sets of species, mostly small numbers (Figure 1), so the comparison of the tree topologies involves a pruning step where the trees are reduced to the overlap in the species sets; in many cases, the species sets do not overlap, so the distance between the corresponding trees cannot be calculated (see Materials and methods). To avoid the uncertainty associated with the pruning procedure and to explore the properties of those few trees that could be considered to represent the 'core of life', we analyzed, along with the complete set of trees, a subset of nearly universal trees (NUTs). As the strictly universal gene core of cellular life is very small and continues to shrink (owing to the loss of generally 'essential' genes in some organisms with small genomes, and to errors of genome annotation) [45,46], we defined NUTs as trees for those COGs that were represented in more than 90% of the included prokaryotes; this definition yielded 102 NUTs. Not surprisingly, the great majority of the NUTs are genes encoding proteins involved in translation and the core aspects of transcription (Additional data file 3). For most of the analyses described below, we analyzed the NUTs in parallel with the complete set of trees in the forest of life or else traced the position of the NUTs in the results of the global analysis; however, this approach does not amount to using the NUTs as an *a priori *standard against which to compare the rest of the trees.

### The NUTs contain a strong, consistent phylogenetic signal, with independent HGT events

**Figure 2:**
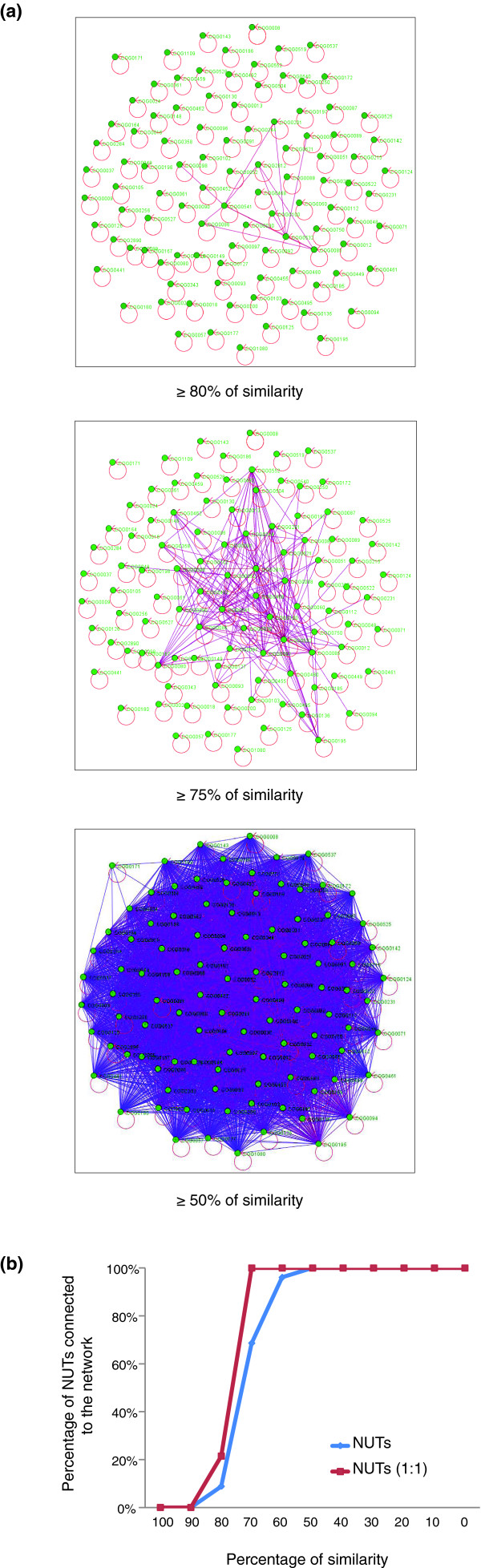
The network of similarities among the nearly universal trees (NUTs). **(a) **Each node (green dot) denotes a NUT, and nodes are connected by edges if the similarity between the respective edges exceeds the indicated threshold. **(b) **The connectivity of 102 NUTs and the 14 1:1 NUTs depending on the topological similarity threshold.

We begin the systematic exploration of the forest of life with the grove of 102 NUTs. Figure 2a shows the network of connections between the NUTs on the basis of topological similarity. The results of this analysis indicated that the topologies of the NUTs were, in general, highly coherent, with a nearly full connectivity reached at 50% similarity ((1 – BSD) × 100) cutoff (BSD is boot split distance; see Materials and methods for details; Figure 2b).

In 56% of the NUTs, archaea and bacteria were perfectly separated, whereas the remaining 44% showed indications of HGT between archaea and bacteria (13% from archaea to bacteria, 23% from bacteria to archaea and 8% in both directions; see Materials and methods for details and Additional data file 3). In the rest of the NUTs, there was no sign of such interdomain gene transfer but there were many probable HGT events within one or both domains (data not shown).

**Figure 3:**
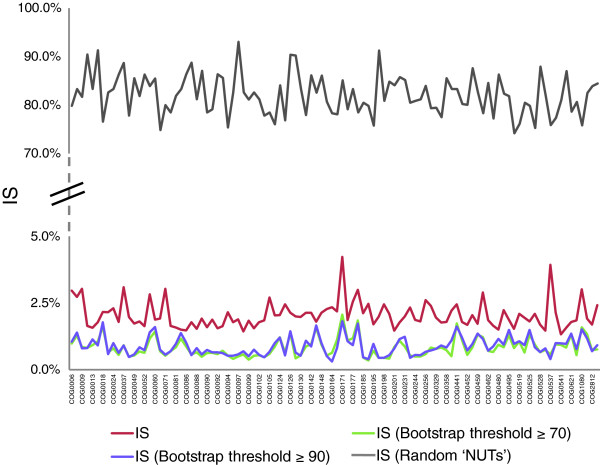
Topological inconsistency of the 102 NUTs compared with random trees of the same size. The NUTs are shown by red lines and ordered by increasing inconsistency score (IS) values. Grey lines show the IS values for the random trees corresponding to each of the NUTs. Each random tree had the same set of species as the corresponding NUT. The IS of each NUT was calculated using as the reference all 102 NUTs and the IS of each random tree was calculated using as the reference all 102 random trees. Also shown are the IS values obtained for those partitions of each NUT that were supported by bootstrap values greater than 70% or less than 90%.

**Figure 4:**
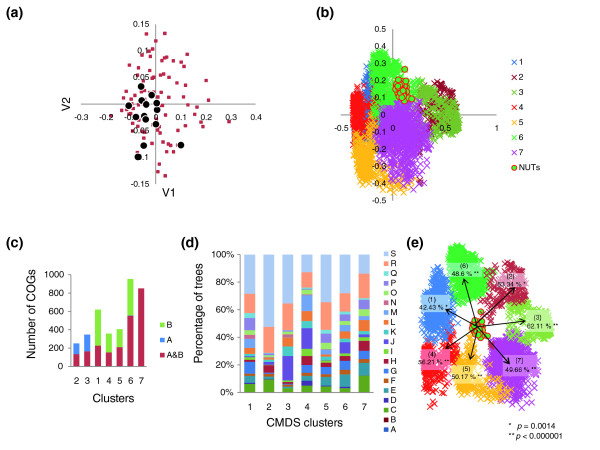
Clustering of the NUTs and the trees in the forest of life using the classical multidimensional scaling (CMDS) method. **(a) **The best two-dimensional projection of the clustering of 102 NUTs (brown squares) in a 30-dimensional space. The 14 1:1 NUTs (corresponding to COGs consisting of 1:1 orthologs) are shown as black circles. V1, V2, variables 1 and 2, respectively. **(b) **The best two-dimensional projection of the clustering of the 3,789 COG trees in a 669-dimensional space. The seven clusters are color-coded and the NUTs are shown by red circles. **(c) **Partitioning of the trees in each cluster between the two prokaryotic domains: blue, archaea-only (A); green, bacteria-only (B); brown, COGs including both archaea and bacteria (A&B). **(d) **Classification of the trees in each cluster by COG functional categories [41,42]: A, RNA processing and modification; B, chromatin structure and dynamics; C, energy transformation; D, cell division and chromosome partitioning; E, amino acid metabolism and transport; F, nucleotide metabolism and transport; G, carbohydrate metabolism and transport; H, coenzyme metabolism and transport; I, lipid metabolism; J, translation and ribosome biogenesis; K, transcription; L, replication and repair; M, cell envelope and outer membrane biogenesis; N, cell motility and secretion; O, post-translational modification, protein turnover, chaperones; P, inorganic ion transport and metabolism; Q, secondary metabolism; R, general functional prediction only; S, uncharacterized. **(e) **The mean similarity values between the 102 NUTs and each of the seven tree clusters in the forest of life (colors as in (b)).

The inconsistency among the NUTs ranged from 1.4 to 4.3%, whereas the mean value of inconsistency for an equal-sized set (102) of randomly generated trees with the same number of species was approximately 80% (Figure 3), indicating that the topologies of the NUTs are highly consistent and non-random. We explored the relationships among the 102 NUTs by embedding them into a 30-dimensional tree space using the CMDS procedure [47,48] (see Materials and methods for details). The gap statistics analysis [49] reveals a lack of significant clustering among the NUTs in the tree space. Thus, all the NUTs seem to belong to a single, unstructured cloud of points scattered around a single centroid (Figure 4a). This organization of the tree space is most compatible with individual trees randomly deviating from a single, dominant topology (the tree of life), apparently as a result of HGT (but possibly also due to random errors in the tree-construction procedure). To further assess the potential contribution of phylogenetic analysis artifacts to observed inconsistencies between the NUTs, we carried out a comparative analysis of these trees with different bootstrap support thresholds (that is, only splits supported by bootstrap values above the respective threshold value were compared). As shown in Figure 3, particularly low IS levels were detected for splits with high-bootstrap support, but the inconsistency was never eliminated completely, suggesting that HGT is a significant contributor to the observed inconsistency among the NUTs.

For most of the NUTs, the corresponding COGs included paralogs in some organisms, so the most conserved paralog was used for tree construction (see Materials and methods for details). However, 14 NUTs corresponded to COGs consisting strictly of 1:1 orthologs (all of them ribosomal proteins). These 1:1 NUTs were similar to others in terms of connectivity in the networks of trees, although their characteristic connectivity was somewhat greater than that of the rest of the NUTs (Figure 2b) or their positions in the single cluster of NUTs obtained using CMDS (Figure 4a), indicating that the selection of conserved paralogs for tree analysis in the other NUTs did not substantially affect the results of topology comparison.

The NUTs include highly conserved genes whose phylogenies have been extensively studied previously. It is not our aim here to compare these phylogenies in detail and to discuss the implications of particular tree topologies. Nevertheless, it is worth noting, by way of a reality check, that the putative HGT events between archaea and bacteria detected here by the separation score analysis (see Materials and methods for details) are compatible with previous observations (Additional data file 3). In particular, HGT was inferred for 83% of the genes encoding aminoacyl-tRNA synthetases (compared with the overall 44%), essential components of the translation machinery that are known for their horizontal mobility [50,51], whereas no HGT was predicted for any of the ribosomal proteins, which belong to an elaborate molecular complex, the ribosome, and hence appear to be non-exchangeable between the two prokaryotic domains [52,53]. In addition to the aminoacyl-tRNA synthetases, and in agreement with many previous observations ([54] and references therein), evidence of HGT between archaea and bacteria was seen also for the majority of the metabolic enzymes that belonged to the NUTs, including undecaprenyl pyrophosphate synthase, glyceraldehyde-3-phosphate dehydrogenase, nucleoside diphosphate kinase, thymidylate kinase, and others (Additional data file 3).

Most of the NUTs, as well as the supertree, also showed a good topological agreement with trees produced by analysis of concatenations of universal proteins [37,55]; notably, the mean distance from the NUTs to the tree of 31 concatenated (nearly) universal proteins [37] was very similar to the mean distance among the 102 NUTs and that between the full set of NUTs and the 14 1:1 NUTs (Table 1). In other words, the 'Universal Tree of Life' constructed by Ciccarelli *et al*. [37] was statistically indistinguishable from the NUTs but did show obvious properties of a consensus topology (the 1:1 ribosomal protein NUTs were more similar to the universal tree than the rest of the NUTs, in part because these proteins were used for the construction of the universal tree and, in part, presumably because of the low level of HGT among ribosomal proteins).

**Table 1:** Distances between the NUTs and the 'universal tree of life'

	TOL	NUTs	NUTs (1:1)	Random NUTs
TOL	0			
NUTs	0.604 ± 0.096	0.659 ± 0.076		
NUTs (1:1)	0.554 ± 0.050	0.639 ± 0.065	0.607 ± 0.065	
Random NUTs	0.994 ± 0.011	0.998 ± 0.004	0.999 ± 0.004	0.998 ± 0.005

The table shows the mean split distance ± standard deviation for the three sets of NUTs and the 'universal tree of life' (TOL) [37]. The overlap between the tree of life and the NUTs consisted of 47 species, so the distances were computed after pruning the NUTs to that set of species.

**Figure 5:**
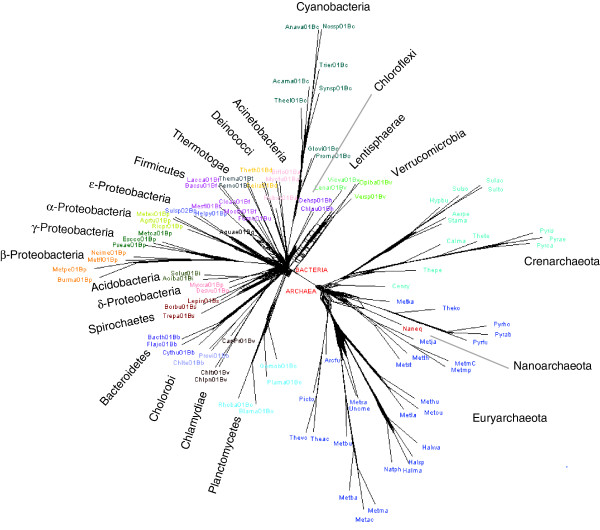
The supernetwork of the NUTs. For spcies abbreviations see Additional File 1.

The overall conclusion on the evolutionary trends among the NUTs is unequivocal. Although the topologies of the NUTs were, for the most part, not identical, so that the NUTs could be separated by their degree of inconsistency (a proxy for the amount of HGT), the overall high consistency level indicated that the NUTs are scattered in the close vicinity of a consensus tree, with the HGT events distributed randomly, at least approximately. Examination of a supernetwork built from the 102 NUTs suggests that the incongruence among these trees is mainly concentrated at the deepest levels (except for the clean archaeal-bacterial split), with a much greater congruence at shallow phylogenetic depths (Figure 5). Of course, one should keep in mind that the unequivocal separation of archaea and bacteria in the supernetwork is obtained despite the apparent substantial interdomain HGT (in around 44% of the NUTs; see above), with the implication that HGT is likely to be even more common between the major branches within the archaeal and bacterial domains. These results are congruent with previous reports on the apparently random distribution of HGT events in the history of highly conserved genes, in particular those encoding proteins involved in translation [29,53], and on the difficulty of resolving the phylogenetic relationships between the major branches of bacteria [28,56,57] and archaea [58,59].

### The NUTs versus the forest of life

We analyzed the structure of the forest of life by embedding the 3,789 COG trees into a 669-dimensional space (see Materials and methods for details) using the CMDS procedure [47,48] (a CMDS analysis of the entire set of 6,901 trees in the forest was beyond the capacity of the R software package used for this analysis; however, the set of COG trees included most of the trees with a large number of species for which the topology comparison is most informative). A gap statistics analysis [49] of K-means clustering of these trees in the tree space did reveal distinct clusters of trees in the forest. The partitioning of the forest into seven clusters of trees (the smallest number of clusters for which the gap function did not significantly increase with the increase of the number of clusters; Figure 4b) produces groups of trees that differed in terms of the distribution of the trees by the number of species, the partitioning of archaea-only and bacteria-only trees, and the functional classification of the respective COGs (Figure 4c,d). For instance, clusters 1, 4, 5 and 6 were enriched for bacterial-only trees, all archaeal-only trees belong to clusters 2 and 3, and cluster 7 consists entirely of mixed archaeal-bacterial clusters; notably, all the NUTs form a compact group inside cluster 6 (Figure 4b). The results of the CMDS clustering support the existence of several distinct 'attractors' in the forest; however, we have to emphasize caution in the interpretation of this clustering because trivial separation of the trees by size could be an important contribution. The approaches to the delineation of distinct 'groves' within the forest merit further investigation. The most salient observation for the purpose of the present study is that all the NUTs occupy a compact and contiguous region of the tree space and, unlike the complete set of the trees, are not partitioned into distinct clusters by the CMDS procedure (Figure 4a).

**Figure 6:**
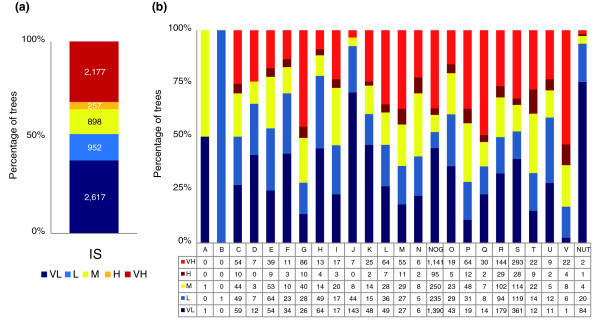
Distribution of the trees in the forest of life by topological inconsistency. **(a) **All trees. **(b) **Trees partitioned into COG functional categories. The data for the NUTs are also shown. The IS values are classified as very low (VL; values less than 40% of mean IS), low (L; values less than 20% of mean IS), medium (M; values around mean IS ± 20%), high (H; more than 20% of mean IS), and very high (VH; values more than 40% of mean IS).

Not unexpectedly, the trees in the forest show a strong signal of numerous HGT events, including interdomain gene transfers. Specifically, in the group of 1,473 trees that include at least five archaeal species and at least five bacterial species, perfect separation of archaea and bacteria was seen in only 13%. This value is the low bound of the fraction of trees that are free of interdomain HGT because, even when archaea and bacteria are perfectly separated, such HGT cannot be ruled out, for instance, in cases when a small, compact archaeal branch is embedded within a bacterial lineage (or vice versa). We further explored the distribution of ISs among the trees. Rather unexpectedly, the majority of the trees (about 70%) had either a very high or a very low level of inconsistency, suggestive of a bimodal distribution of the level of HGT (Figure 6a). Furthermore, the distribution of the ISs across functional classes of genes was distinctly non-random: some categories, in particular, all those related to transcription and translation, but also some classes of metabolic enzymes, were strongly enriched in trees with very low ISs, whereas others, such as genes for enzymes of carbohydrate metabolism or proteins involved in inorganic ion transport, were characterized by very high inconsistency (Figure 6b). The great majority of the NUTs that include, primarily, genes for proteins involved in translation have very low ISs (Figure 6b). These observations, in part, overlap with the predictions of the well-known complexity hypothesis [52], according to which the rate of HGT is low for those genes that encode subunits of large macromolecular complexes, such as the ribosome, and much higher for those genes whose products do not form such complexes. However, some of the findings reported here, such as the very low inconsistency values among genes for enzymes of nucleotide and coenzyme biosynthesis, do not readily fit the framework of the complexity hypothesis.

**Figure 7:**
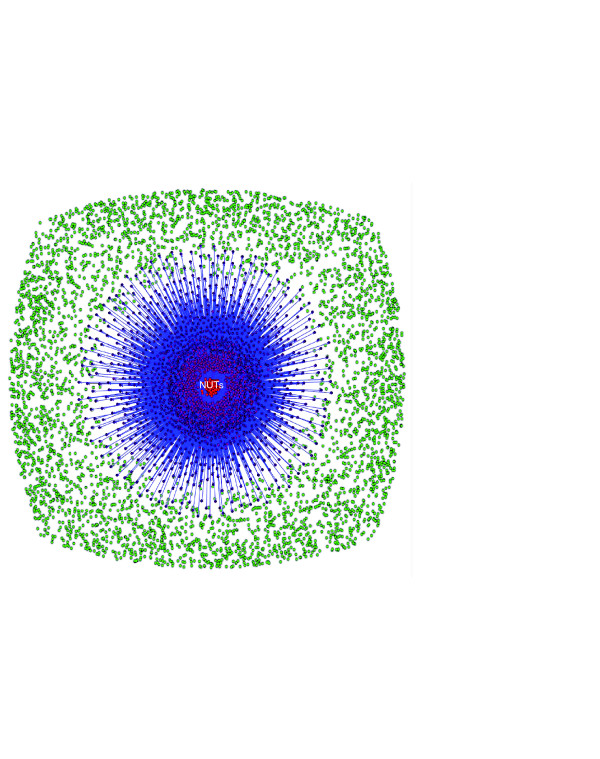
Network representation of the 6,901 trees of the forest of life. The 102 NUTs are shown as red circles in the middle. The NUTs are connected to trees with similar topologies: trees with at least 50% of similarity with at least one NUT (*P*-value < 0.05) are shown as purple circles and connected to the NUTs. The rest of the trees are shown as green circles.

**Figure 8:**
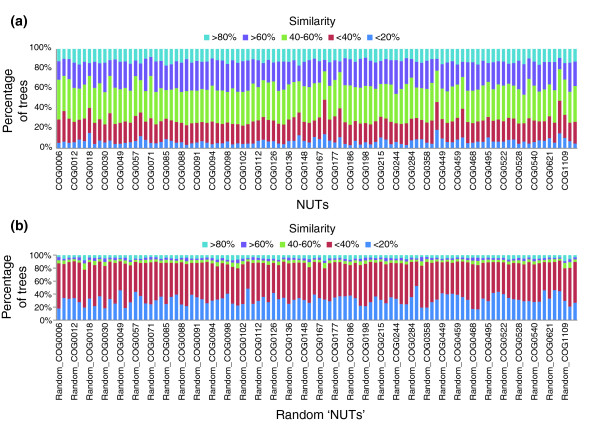
Similarity of the trees in the forest of life to the NUTs. **(a) **For each of the 102 NUTs, the breakdown of the rest of the trees in the forest by percent similarity is shown. **(b) **The same breakdown for 102 random trees generated from the NUTs.

We constructed a network of all 6,901 trees that collectively comprise the forest and examined the position and the connectivity of the 102 NUTs in this network (Figure 7). At the 50% similarity cutoff and a *P*-value < 0.05, the 102 NUTs were connected to 2,615 trees (38% of all trees in the forest; Figure 7), and the mean similarity of the trees to the NUTs was approximately 50%, with similar distributions of strongly, moderately and weakly similar trees seen for most of the NUTs (Figure 8a). In sharp contrast, using the same similarity cutoff, 102 randomized NUTs were connected to only 33 trees (about 0.5% of the trees) and the mean similarity to the trees in the forest was approximately 28%. Accordingly, the random trees showed completely different distributions of similarity to the trees in the forest, with the consistent predominance of moderately and weakly similar trees (Figure 8b). These findings emphasize the highly non-random topological similarity between the NUTs and a large part of the forest of life, and show that this similarity is not an artifact of the large number of species in the NUTs.

A comparison between the NUTs and the seven clusters revealed by the CMDS analysis also showed comparable average levels of similarity (close to 50%) to each of the clusters (Figure 4e). Considering this relatively high and uniform level of connectivity between the NUTs and the rest of the trees in the forest, and the lack of a pronounced structure within the set of the NUTs themselves (see above), it appears that the NUTs potentially could be a reasonable representation of a central trend in the forest of life, despite the apparent existence of distinct 'groves' and the high prevalence of HGT.

### The dependence of tree inconsistency on the phylogenetic depth

**Figure 9:**
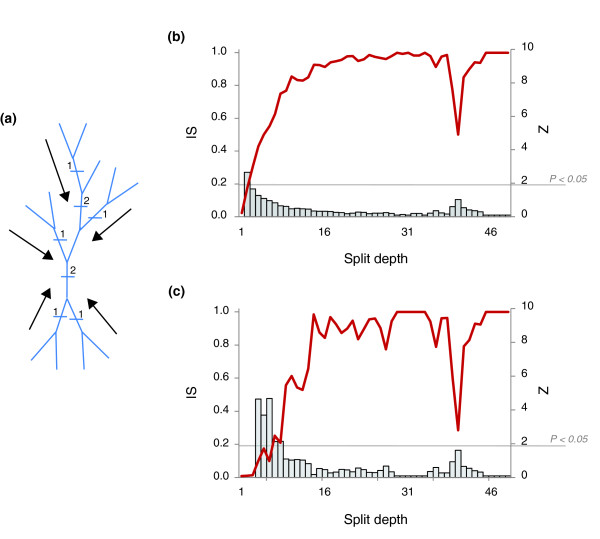
The dependence of tree inconsistency on the split depth. The mean inconsistency value (IS) is shown for each split depth (1 to 46), which was determined by counting the splits in the trees from leaves to the center of the tree. **(a) **Schematic of the procedure used to determine the split depth. **(b) **IS plotted against split depth for all 6,901 trees of the forest of life. **(c) **IS plotted against split depth for the 102 NUTs. The vertical axis on the right in (b, c) shows the z-score, and the grey bars show the z-score values for the respective depths.

An important issue that could potentially affect the status of the NUTs as a representation of a central trend in the forest of life is the dependence of the inconsistency between trees on the phylogenetic depth. As suggested by the structure of the supernetwork of the NUTs (Figure 4), the inconsistency of the trees notably increased with phylogenetic depth. We examined this problem quantitatively by tallying the IS values separately for each depth (the split depth that was determined by counting splits from the leaves to the center of the tree; see Materials and methods; Figure 9a) and found that the inconsistency of the forest was substantially lower than that of random trees at the top levels but did not significantly differ from the random values at greater depths (Figure 9b). The only deep signal that was apparent within the entire forest was seen at depth 40 and corresponded to the split between archaea and bacteria (Figure 9b); when only the NUTs were similarly analyzed, an additional signal was seen at depth 12, which corresponds to the separation between Crenarchaeota and Euryarchaeota (Figure 9c). These findings indicate that most of the edges that support the network of trees are based on the congruence of the topologies in the crowns of trees whereas the deep splits are, mostly, inconsistent. Together with a previous report that the congruence between phylogenetic trees of conserved prokaryotic proteins at deep levels is no greater than random [57], these findings cast doubt on the feasibility of identification of a central trend in the forest that could qualify as a tree of life.

### Testing the Biological Big Bang model

The sharply increasing inconsistency at the deep levels of the forest of life suggests the possibility that the evolutionary processes that were responsible for the formation of this part of the forest could be much different from those that were in operation at lesser phylogenetic depths. More specifically, we considered two models of early evolution at the level of archaeal and bacterial phyla: a compressed cladogenesis (CC) model, whereby there is a tree structure even at the deepest levels but the internal branches are extremely short [39]; and a Biological Big Bang (BBB) model under which the early phase of evolution involved horizontal gene exchange so intensive that there is no signal of vertical inheritance in principle [36].

**Figure 10:**
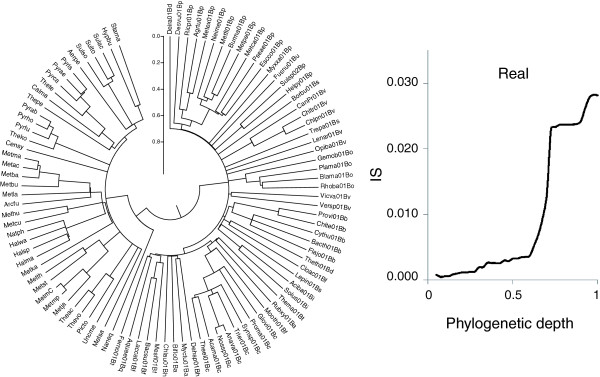
Ultrametric tree produced from the supertree of the 102 NUTs (left) and the dependence of mean inconsistency on phylogenetic depth in this tree (right). The inconsistency versus depth plot is for all 6,901 trees in the forest of life. Species abbreviations as in Figure 5.

**Figure 11:**
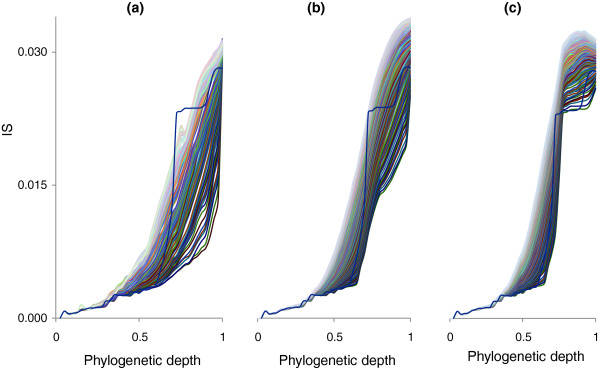
Evolutionary simulations of a Biological Big Bang at different phylogenetic depths and with different numbers of HGT events. Each panel is a plot of the mean tree inconsistency versus phylogenetic depth (in the ultrametric tree). The empirical dependence is shown by a thick blue line, and the results of simulations with 1 to 200 HGT events are shown by thin lines along a color gradient. **(a) **BBB simulated at depth 0.6; **(b) **BBB simulated at depth 0.7; **(c) **BBB simulated at depth 0.8.

**Figure 12:**
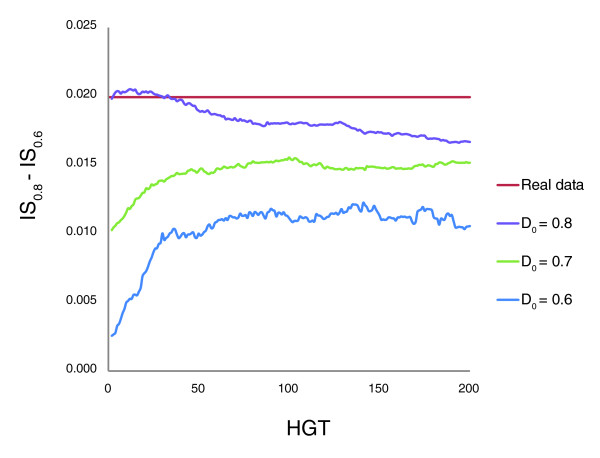
Drop in IS values between phylogenetic depths of 0.6 and 0.8 for the real data and three simulations of the Biological Big Bang (BBB). Red, real data; blue, BBB simulated at the depth of 0.6; green, BBB simulated at the depth of 0.7; violet, BBB simulated at the depth of 0.8. The horizontal axis shows the number of simulated HGT events and the vertical axis shows the differences between IS values at the phylogenetic depths of 0.8 and 0.6.

We simulated the evolutionary processes that produced the forest of life under each of these models. To this end, it was necessary to represent the phylogenetic depth as a continuous value that would be comparable between different branches (as opposed to the discrete levels unique for each tree that were used to generate the plots in Figure 9). This task was achieved using an ultrametric tree that was produced from the supertree of the 102 NUTs (see Materials and methods; Figure 10). The inconsistency of the forest of life sharply increases, in a phase-transition-like fashion, between the depths of 0.7 and 0.8 (Figure 10). We attempted to fit this empirically observed curve with the respective curves produced by simulating the BBB at different phylogenetic depths by randomly shuffling the tree branches at the given depth and modeling the subsequent evolution as a tree-like process with different numbers of HGT events. The results indicate that only by simulating the BBB at the depth of 0.8 could a good fit with the empirical curve be reached (Figures 11c and 12). This depth is below the divergence of the major bacterial and archaeal phyla (Figure 10). Simulation of the BBB at the critical depth of 0.7 or above (completely erasing the phylogenetic signal below the phylum level) did not yield a satisfactory fit (Figures 11a,b and 12), suggesting that the CC model is a more appropriate representation of the early phases of evolution of archaea and bacteria than the BBB model. In other words, the signal of vertical inheritance (a central trend in the forest of life) is detectable even at these phylogenetic depths, although given the high level of inconsistency, the determination of the correct tree topology of the deepest branches in the tree is problematic at best. The results of this analysis do not rule out the BBB model as the generative mechanism underlying the divergence of archaea and bacteria, but this scenario cannot be tested in the manner described above because of the absence of an outgroup. Effectively, simulation of a BBB at a depth of 0.8 or greater is meaningless within the context of the present analysis or any imaginable further analysis, because the archaea and bacteria are thought to be the primary lineages in the evolution of life on Earth.

**Figure 13:**
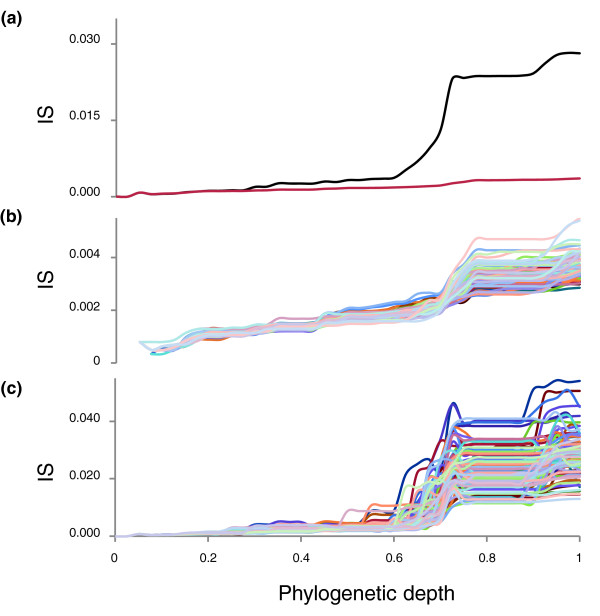
Drop in IS values between phylogenetic depths of 0.6 and 0.8 for the real data and three simulations of the Biological Big Bang (BBB). Red, real data; blue, BBB simulated at the depth of 0.6; green, BBB simulated at the depth of 0.7; violet, BBB simulated at the depth of 0.8. The horizontal axis shows the number of simulated HGT events and the vertical axis shows the differences between IS values at the phylogenetic depths of 0.8 and 0.6.

Finally, when we compared the dependence of the inconsistency on phylogenetic depth for the 102 NUTs and the complete FOL, the NUTs showed a comparable level of inconsistency at low depths but did not display the sharp transition at greater depths, so that below the transition (the CC phase of evolution) seen in the forest of life, the inconsistency of the NUTs was approximately tenfold lower (Figure 13). These results emphasize the relatively strong (compared to the rest of the trees in the forest) vertical signal that is present in the NUTs throughout the entire range of phylogenetic depths.

## Conclusion

Recent developments in prokaryotic genomics reveal the omnipresence of HGT in the prokaryotic world and are often considered to undermine the tree of life concept – uprooting the tree of life [9,11,22,35,60]. There is no doubt that the now well-established observations that HGT spares virtually no genes at some stages in their history [15,16] overthrow a 'strong' tree of life concept under which all (or the substantial majority) of the genes would tell a consistent story of genome evolution (the species tree, or the tree of life) if analyzed using appropriate methods. However, is there any hope of salvaging the tree of life as a statistical central trend [28]? The results of a comprehensive comparative analysis of phylogenetic trees for prokaryotic genes described here suggest a positive answer to this crucial question.

The message from this analysis is twofold. On the one hand, we detected high levels of inconsistency among the trees comprising the forest of life, most probably due to extensive HGT, a conclusion that is supported by more direct observations of numerous probable transfers of genes between archaea and bacteria. On the other hand, we detected a distinct signal of a consensus topology that was particularly strong in the NUTs. Although the NUTs showed a substantial amount of apparent HGT, the transfer events seemed to be distributed randomly and did not obscure the vertical signal. Moreover, the topology of the NUTs was quite similar to those of numerous other trees in the forest, so although the NUTs certainly cannot represent the forest completely, this set of largely consistent, nearly universal trees is a reasonable candidate for representing a central trend. However, the opposite side of the coin is that the consistency between the trees in the forest is high at shallow depths of the trees and abruptly drops, almost down to the level of random trees, at greater phylogenetic depths that correspond to the radiation of archaeal and bacterial phyla. This observation casts doubt on the existence of a central trend in the forest of life and suggests the possibility that the early phases of evolution might have been non-tree-like (a Biological Big Bang [36]). To address this problem directly, we simulated evolution under the CC model [39,40] and under the BBB model, and found that the CC scenario better approximates the observed dependence between tree inconsistency and phylogenetic depth. Thus, a consistent phylogenetic signal seems to be discernible throughout the evolution of archaea and bacteria but, under the CC model, the prospect of unequivocally resolving the relationships between the major archaeal and bacterial clades is bleak.

The most straightforward interpretation of the detected central trend in the forest of life is that it represents vertical inheritance permeating the entire history of archaea and bacteria. A contribution from 'highways' of HGT (that is, preferential HGT between certain groups of archaea and bacteria) that could mimic vertical evolution [15] cannot be ruled out. However, in our view, the lack of significant clustering within the group of NUTs and the comparable high levels of similarity between the NUTs and different clusters of trees in the forest suggest that the trend, even if relatively weak, is primarily vertical.

In summary, HGT is pervasive in the prokaryotic world, so that there are very few fully consistent NUTs. Thus, the original tree of life concept is obsolete: it would not even be a 'tree of one percent' [38]. Nevertheless, there seems to be a discernible signal of consistency between the trees in the forest of life, down to the deepest branching levels. Whether or not this central trend is denoted a tree of life could be a matter of convention and convenience, but the nature of this trend as well as the other trends that can be discerned in the forest merit further investigation.

## Materials and methods

### Clusters of orthologous genes for phylogenetic tree analysis

The analyzed dataset consisted of representatives of 6,901 clusters of likely orthologs from the COGs database [41,42] or the EggNOG database [43] from 100 prokaryotic species – 59 bacteria and 41 archaea – that were manually selected to represent all the major divisions of the two prokaryotic domains (Additional data file 1). The BeTs algorithm [41] was used to identify the orthologs with the highest mean similarity to the other members of a cluster ('index' orthologs [61]), so that each of the final clusters contained a maximum of 100 sequences (no more than one from each of the included organisms). The rationale behind the selection of index orthologs for phylogenetic analysis is that this procedure identifies the members of co-orthologous gene sets that experienced minimal (if any) acceleration of evolution as a result of gene duplication, and accordingly minimizes the potential long-branch artifacts. A group of 102 COGs that were represented in more than 90 organisms was defined as the subset of NUTs (Additional data file 3). Finally, 12 COGs containing more than 300 sequences each were excluded from the subsequent analysis.

### Protein sequence alignment and tree construction

The protein sequences from each COG were aligned using the Muscle program [62] with default parameters and all alignments were refined using the Gblocks program [63] with the minimal length of a block set at six amino acid positions, and the maximum number of allowed contiguous non-conserved amino acid positions set at 20. The maximum likelihood phylogenetic trees were constructed under the best substitution model using the Multiphyl program, which was also used for bootstrap analysis [64]. The Multiphyl program employs methods from the ModelGenerator program to choose, for each alignment, the best of 88 models of amino acid substitution [65]. The entire set of 6,901 trees used in this study is contained in Additional data file 2, and all alignments used for the tree construction are available at [66].

### Supernetwork construction and analysis

The phylogenetic supernetwork from the 102 NUTs was built following the method developed by Huson *et al*. [67] and implemented in the SplitsTree4 program [68] with default parameters. The supernetwork was used for an initial overview of the 102 NUTs set to identify signals and incongruence at different phylogenetic depths. The signals identified by the examination of the supernetwork were verified by the comparative analysis of the tree topologies and by the calculation of the IS against the phylogenetic depth.

### Ultrametric tree

The topology of the ultrametric tree was obtained from the supertree of the 102 NUTs using the CLANN program [69]. The branch lengths from each of the 6,901 trees was used to calculate the average distance between each pair of species. The matrix obtained was used to calculate the branch lengths of the supertree. This supertree with branch lengths was then used to construct an ultrametric tree using the program KITSCH from the Phylip package [70] and rescaled to a depth range of 0 to 1. This tree was used to compute phylogenetic depth in the analysis of the dependence of tree inconsistency on phylogenetic depth.

### Tree comparison

An all-against-all comparison of the trees was performed using a new method that we denoted BSD. The BSD method is a modification of the split distance (SD) method for tree comparison [71] that additionally takes into account the bootstrap values of the trees. Both indices range from 0 to 1 but the SD method assigns equal weights to all branches in a tree, whereas under the BSD method the distance between two trees depends on the level of bootstrap support for the branches of each tree. The BSD corresponds to the average [*BSD *= (*eBSD *+ *dBSD*)/2] of the BSD of equal splits between two trees (*eBSD *= 1 - [(*e*/*a*)·*x*]) and the BSD of the different splits (*dBSD *= (*d*/*a*)·*y*). Here *e *is the sum of bootstrap values of equal splits, *d *is the sum of bootstrap values of different splits, *a *is the sum of the bootstrap values of all splits, *x *is the mean bootstrap value of equal splits, and *y *is the mean bootstrap value of different splits.

The pairwise comparison was made for trees with leaf sets that either completely or partially overlap. If trees partially overlap in at least four species, they are pruned to their common leaf set in order to compare the topologies. If two trees cannot be compared because they overlap by fewer than four species, a maximum BSD of 1 was assigned.

### Classical multidimensional scaling analysis

CMDS, also known as principal coordinate analysis, embeds *n *data points implied by a [*n *× *n*] distance matrix into an *m*-dimensional space (*m *<*n*) in such a manner that, for any *k *∈ [1, *m*], the embedding into the first *k *dimensions is the best in terms of preserving the original distances between the points [47,48]. Given that in this work the relationships between phylogenetic trees are defined in terms of tree-to-tree distance, CMDS is the natural approach to analyze the structure of the tree space. The function cmdscale of the R package was used to perform CMDS on BSD distances between the trees. The number of dimensions corresponding to preserving 75% of the total inertia (30 dimensions for 102 NUTs and 669 dimensions for 3,789 COG trees) was chosen for further analysis.

Clustering of data points in multidimensional space was performed using the kmeans function of the R package that implements the K-means algorithm [72]. The choice of the optimal number of clusters was performed using an R script implementing the gap statistics algorithm [49]. In the case of the 102 NUTs, the highest value of the gap function was observed at *K *= 1, for *K *∈ [1,30], indicating a single cluster in the tree space. In the case of the 3,789 COG trees, the gap function was increasing for *K *∈ [1,30], suggesting a strong tendency of these trees to form multiple clusters. Following the recommendations of Tibshirani *et al*. [49], *K *= 7 was chosen as the lowest number of clusters for which the value of the gap function for *K *= *k *+ 1 was not significantly higher than that for *K *= *k *(z-score below 1.96, corresponding to 0.05 significance level).

### Inference of horizontal gene transfer

To analyze all possible cases of HGT between bacteria and archaea in the NUTs, we used the score of separation *B/A *(*SS*_*B*/*A*_) that was calculated, for each branch in a tree, by subtracting the number of bacteria or archaea on one side of the tree from the number of bacteria or archaea on the other side (*SS*_*B*/*A *_= |*pA*_*left*_*-pA*_*right*_| = |*pB*_*left*_*-pB*_*right*_|) where *pA *and *pB *are the percentages of archaeal and bacterial species, respectively. The tree was assigned the highest value of the separation score obtained for all its branches. This score was also used to analyze possible cases of HGT between bacteria and archaea in those trees that include at least five archaeal species and at least five bacterial species.

The value of the *B/A *score ranges from 0 to 1. A tree is considered free of archaeal-bacterial HGT if the *B/A *score equals 1, that is, archaea and bacteria are perfectly separated in the given tree. The *B/A *score values of less than 1 are considered indicative of HGT. These cases can be classified into three categories: first, HGT from bacteria to archaea (B → A) when there is a nearly perfect separation of these two groups but inside the bacteria there is a small group of archaeal species; second, HGT from archaea to bacteria (A → B) when there is a small group of bacterial species inside the archaeal domain; and third, bidirectional HGT events (A ↔ B) when the greatest score of separation *B/A *is obtained by mixing archaeal and bacterial species (*pA*_*left*_*, pA*_*right*_*, pB*_*left *_and *pB*_*right *_<100%).

### Inconsistency score

IS is the fraction of the times that the splits from a given tree are found in all *N *trees that comprise the forest of life: *IS *= [(1/*Y *- *IS*_*min*_]/*IS*_*max*_, where *X *is the number of splits in the given tree, and *Y *is the number of times the splits from the given tree are found in all trees of the forest. Under this formula, *IS*_*min *_= 1/(*XN*) and *IS*_*max *_= [1/(*X*)] - *IS*_*min*_. Thus, IS is a measure of how representative the topology of the given tree is of the entire forest of life.

### Split depth and phylogenetic depth

The IS was calculated along the depth of the trees, namely, split depth and phylogenetic depth. The split depth was calculated for each phylogenetic tree according to the number of splits from the tips to the center of the tree. The value of split depth ranged from 1 (2 species – 1) to 49 ((100 species/2) – 1). The phylogenetic depth was obtained from the branch lengths of the rescaled ultrametric tree and ranged from 0 to 1.

### Simulation of Biological Big Bang and HGT

The simulation of a BBB was performed by cutting the ultrametric tree at different levels of depth (*D*_0_) and reassembling the bottom part of the tree to simulate infinite numbers of HGT events. The BBB simulation was made at *D*_0 _= 0.6, *D*_0 _= 0.7 and *D*_0 _= 0.8, and repeated 100 times each. The different levels of depth simulated are *D*_0 _= 0.6, corresponding to the depth just after the hypothetical BBB, that is, in the hypothetical tree-like phase; *D*_0 _= 0.7, which corresponds to the hypothetical BBB; and *D*_0 _= 0.8, which corresponds to the hypothetical biological inflation phase. Each tree obtained after the simulation of the BBB was processed to simulate an increasing number of HGT events from 1 to 200. These HGT simulations were performed by cutting the tree at random depth *D*_*R *_(*D*_*R *_<*D*_0_) and swapping a random pair of branches.

## Additional data files

Additional data file 1 contains a list of species (59 bacterial and 41 archaeal) used for the FOL construction. Additional data file 2 contains all the phylogenetic trees. Additional data file 3 contains a list of the 102 COGs that are represented in at least 90 of the100 selected archaea and bacteria.

## Supplementary Material

Additional data file 1 A list of species (59 bacterial and 41 archaeal) used for the FOL construction. Click here for file

Additional data file 2 All the phylogenetic trees Click here for file

Additional data file 3 A list of the 102 COGs that are represented in at least 90 of the100 selected archaea and bacteria. Click here for file
