# Supplementary material for: Search for a 'Tree of Life' in the thicket of the phylogenetic forest
Source: J Biol. 2009 Jul 13;8(6):59. doi: 10.1186/jbiol159 (PMC2737373; doi:10.1186/jbiol159)
Supplement: Additional data file 1 — A list of species (59 bacterial and 41 archaeal) used for the FOL construction. [file jbiol159-S1.doc]

**List of species (59 Bacteria and 41 Archaea) used for the FOL construction**

| Censy | *Cenarchaeum symbiosum* | Archaea | Crenarchaeota | Cenarchaeales |
| --- | --- | --- | --- | --- |
| Aerpe | *Aeropyrum pernix* | Archaea | Crenarchaeota | Desulfurococcales |
| Hypbu | *Hyperthermus butylicus* | Archaea | Crenarchaeota | Desulfurococcales |
| Stama | *Staphylothermus marinus F1* | Archaea | Crenarchaeota | Desulfurococcales |
| Sulac | *Sulfolobus acidocaldarius DSM 639* | Archaea | Crenarchaeota | Sulfolobales |
| Sulso | *Sulfolobus solfataricus* | Archaea | Crenarchaeota | Sulfolobales |
| Sulto | *Sulfolobus tokodaii* | Archaea | Crenarchaeota | Sulfolobales |
| Calma | *Caldivirga maquilingensis IC-167* | Archaea | Crenarchaeota | Thermoproteales |
| Pyrae | *Pyrobaculum aerophilum* | Archaea | Crenarchaeota | Thermoproteales |
| Pyrca | *Pyrobaculum calidifontis JCM 11548* | Archaea | Crenarchaeota | Thermoproteales |
| Pyris | *Pyrobaculum islandicum DSM 4184* | Archaea | Crenarchaeota | Thermoproteales |
| Thepe | *Thermofilum pendens Hrk 5* | Archaea | Crenarchaeota | Thermoproteales |
| Thete | *Thermoproteus tenax* | Archaea | Crenarchaeota | Thermoproteales |
| Uncme | *Uncultured methanogenic archaeon* | Archaea | Euryarchaeota | ? |
| Arcfu | *Archaeoglobus fulgidus* | Archaea | Euryarchaeota | Archaeoglobales |
| Halma | *Haloarcula marismortui ATCC 43049* | Archaea | Euryarchaeota | Halobacteriales |
| Halsp | *Halobacterium sp* | Archaea | Euryarchaeota | Halobacteriales |
| Halwa | *Haloquadratum walsbyi* | Archaea | Euryarchaeota | Halobacteriales |
| Natph | *Natronomonas pharaonis* | Archaea | Euryarchaeota | Halobacteriales |
| Metth | *Methanobacterium thermoautotrophicum* | Archaea | Euryarchaeota | Methanobacteriales |
| Metst | *Methanosphaera stadtmanae* | Archaea | Euryarchaeota | Methanobacteriales |
| Metja | *Methanococcus jannaschii* | Archaea | Euryarchaeota | Methanococcales |
| MetmC | *Methanococcus maripaludis C5* | Archaea | Euryarchaeota | Methanococcales |
| Metmp | *Methanococcus maripaludis S2* | Archaea | Euryarchaeota | Methanococcales |
| Metla | *Methanocorpusculum labreanum Z* | Archaea | Euryarchaeota | Methanomicrobiales |
| Metcu | *Methanoculleus marisnigri JR1* | Archaea | Euryarchaeota | Methanomicrobiales |
| Methu | *Methanospirillum hungatei JF-1* | Archaea | Euryarchaeota | Methanomicrobiales |
| Metka | *Methanopyrus kandleri* | Archaea | Euryarchaeota | Methanopyrales |
| Metbu | *Methanococcoides burtonii DSM 6242* | Archaea | Euryarchaeota | Methanosarcinales |
| Metsa | *Methanosaeta thermophila PT* | Archaea | Euryarchaeota | Methanosarcinales |
| Metac | *Methanosarcina acetivorans* | Archaea | Euryarchaeota | Methanosarcinales |
| Metba | *Methanosarcina barkeri fusaro* | Archaea | Euryarchaeota | Methanosarcinales |
| Metma | *Methanosarcina mazei* | Archaea | Euryarchaeota | Methanosarcinales |
| Pyrab | *Pyrococcus abyssi* | Archaea | Euryarchaeota | Thermococcales |
| Pyrfu | *Pyrococcus furiosus* | Archaea | Euryarchaeota | Thermococcales |
| Pyrho | *Pyrococcus horikoshii* | Archaea | Euryarchaeota | Thermococcales |
| Theko | *Thermococcus kodakaraensis KOD1* | Archaea | Euryarchaeota | Thermococcales |
| Picto | *Picrophilus torridus DSM 9790* | Archaea | Euryarchaeota | Thermoplasmales |
| Theac | *Thermoplasma acidophilum* | Archaea | Euryarchaeota | Thermoplasmales |
| Thevo | *Thermoplasma volcanium* | Archaea | Euryarchaeota | Thermoplasmales |
| Naneq | *Nanoarchaeum equitans* | Archaea | Nanoarchaeota | ? |
| Aciba01Bi | *Acidobacteria bacterium Ellin345* | Bacteria | Acidobacteria | Acidobacteriales |
| Solus01Bi | *Solibacter usitatus Ellin6076* | Bacteria | Acidobacteria | Solibacterales |
| Myctu01Ba | *Mycobacterium tuberculosis H37Rv* | Bacteria | Actinobacteria | Actinomycetales |
| Biflo01Ba | *Bifidobacterium longum NCC2705* | Bacteria | Actinobacteria | Bifidobacteriales |
| Rubxy01Ba | *Rubrobacter xylanophilus DSM 9941* | Bacteria | Actinobacteria | Rubrobacterales |
| Aquae01Bq | *Aquifex aeolicus VF5* | Bacteria | Aquificae | Aquificales |
| Bacth01Bb | *Bacteroides thetaiotaomicron VPI-5482* | Bacteria | Bacteroidetes | Bacteroidales |
| Flajo01Bb | *Flavobacterium johnsoniae UW101* | Bacteria | Bacteroidetes | Flavobacteriales |
| Cythu01Bb | *Cytophaga hutchinsonii ATCC 33406* | Bacteria | Bacteroidetes | Sphingobacteriales |
| CanPr01Bv | *Candidatus Protochlamydia amoebophila UWE25* | Bacteria | Chlamydiae | Chlamydiales |
| Chltr01Bv | *Chlamydia trachomatis D/UW-3/CX* | Bacteria | Chlamydiae | Chlamydiales |
| Chlpn01Bv | *Chlamydophila pneumoniae AR39* | Bacteria | Chlamydiae | Chlamydiales |
| Chlte01Bb | *Chlorobium tepidum TLS* | Bacteria | Chlorobi | Chlorobiales |
| Provi01Bb | *Prosthecochloris vibrioformis DSM 265* | Bacteria | Chlorobi | Chlorobiales |
| Chlau01Bh | *Chloroflexus aurantiacus J-10-fl* | Bacteria | Chloroflexi | Chloroflexales |
| Dehsp01Bh | *Dehalococcoides sp BAV1* | Bacteria | Chloroflexi | Dehalococcoidetes |
| Synsp01Bc | *Synechocystis sp PCC 6803* | Bacteria | Cyanobacteria | Chroococcales |
| Theel01Bc | *Thermosynechococcus elongatus BP-1* | Bacteria | Cyanobacteria | Chroococcales |
| Glovi01Bc | *Gloeobacter violaceus PCC 7421* | Bacteria | Cyanobacteria | Gloeobacterales |
| Anava01Bc | *Anabaena variabilis ATCC 29413* | Bacteria | Cyanobacteria | Nostocales |
| Nossp01Bc | *Nostoc sp PCC 7120* | Bacteria | Cyanobacteria | Nostocales |
| Trier01Bc | *Trichodesmium erythraeum IMS101* | Bacteria | Cyanobacteria | Oscillatoriales |
| Proma01Bc | *Prochlorococcus marinus subsp marinus str CCMP1375* | Bacteria | Cyanobacteria | Prochlorales |
| Acama01Bc | *Acaryochloris marina MBIC11017* | Bacteria | Cyanobacteria | unclassified |
| Deira01Bd | *Deinococcus radiodurans R1* | Bacteria | Deinococci | Deinococcales |
| Theth01Bd | *Thermus thermophilus HB27* | Bacteria | Deinococci | Thermales |
| Bacsu01Bf | *Bacillus subtilis subsp subtilis str 168* | Bacteria | Firmicutes | Bacillales |
| Cloac01Bf | *Clostridium acetobutylicum ATCC 824* | Bacteria | Firmicutes | Clostridiales |
| Mesfl01Bf | *Mesoplasma florum L1* | Bacteria | Firmicutes | Entomoplasmatales |
| Lacca01Bf | *Lactobacillus casei ATCC 334* | Bacteria | Firmicutes | Lactobacillales |
| Mooth01Bf | *Moorella thermoacetica ATCC 39073* | Bacteria | Firmicutes | Thermoanaerobacteriales |
| Fusnu01Bu | *Fusobacterium nucleatum subsp nucleatum ATCC 25586* | Bacteria | Fusobacteria | Fusobacteriales |
| Lenar01Bv | *Lentisphaera araneosa HTCC2155* | Bacteria | Lentisphaerae | Lentisphaerales |
| Vicva01Bv | *Victivallis vadensis ATCC BAA-548* | Bacteria | Lentisphaerae | Victivallales |
| Blama01Bo | *Blastopirellula marina DSM 3645* | Bacteria | Planctomycetes | Planctomycetales |
| Gemob01Bo | *Gemmata obscuriglobus* | Bacteria | Planctomycetes | Planctomycetales |
| Plama01Bo | *Planctomyces maris DSM 8797* | Bacteria | Planctomycetes | Planctomycetales |
| Rhoba01Bo | *Rhodopirellula baltica SH 1* | Bacteria | Planctomycetes | Planctomycetales |
| Agrtu01Bp | *Agrobacterium tumefaciens str C58* | Bacteria | Proteobacteria-Alpha | Rhizobiales |
| Metex01Bp | *Methylobacterium extorquens PA1* | Bacteria | Proteobacteria-Alpha | Rhizobiales |
| Ricpr01Bp | *Rickettsia prowazekii str Madrid E* | Bacteria | Proteobacteria-Alpha | Rickettsiales |
| Burma01Bp | *Burkholderia mallei ATCC 23344* | Bacteria | Proteobacteria-Beta | Burkholderiales |
| Metpe01Bp | *Methylibium petroleiphilum PM1* | Bacteria | Proteobacteria-Beta | Burkholderiales |
| Metfl01Bp | *Methylobacillus flagellatus KT* | Bacteria | Proteobacteria-Beta | Methylophilales |
| Neime01Bp | *Neisseria meningitidis MC58* | Bacteria | Proteobacteria-Beta | Neisseriales |
| Desvu01Bp | *Desulfovibrio vulgaris subsp vulgaris str Hildenborough* | Bacteria | Proteobacteria-Delta | Desulfovibrionales |
| Myxxa01Bp | *Myxococcus xanthus DK 1622* | Bacteria | Proteobacteria-Delta | Myxococcales |
| Helpy01Bp | *Helicobacter pylori 26695* | Bacteria | Proteobacteria-Epsilon | Campylobacterales |
| Sulsp02Bp | *Sulfurovum sp NBC37-1* | Bacteria | Proteobacteria-Epsilon | unclassified |
| Escco01Bp | *Escherichia coli K12* | Bacteria | Proteobacteria-Gamma | Enterobacteriales |
| Metca01Bp | *Methylococcus capsulatus str Bath* | Bacteria | Proteobacteria-Gamma | Methylococcales |
| Pseae01Bp | *Pseudomonas aeruginosa PAO1* | Bacteria | Proteobacteria-Gamma | Pseudomonadales |
| Borbu01Bs | *Borrelia burgdorferi B31* | Bacteria | Spirochaetes | Spirochaetales |
| Lepin01Bs | *Leptospira interrogans serovar Copenhageni str Fiocruz L1-130* | Bacteria | Spirochaetes | Spirochaetales |
| Trepa01Bs | *Treponema pallidum subsp pallidum str Nichols* | Bacteria | Spirochaetes | Spirochaetales |
| Ferno01Bt | *Fervidobacterium nodosum Rt17-B1* | Bacteria | Thermotogae | Thermotogales |
| Thema01Bt | *Thermotoga maritima MSB8* | Bacteria | Thermotogae | Thermotogales |
| Opiba01Bv | *Opitutaceae bacterium TAV2* | Bacteria | Verrucomicrobia | Opitutales |
| Versp01Bv | *Methylokorus infernorum V4* | Bacteria | Verrucomicrobia | Verrucomicrobiales |
