# Supplementary material for: Search for a 'Tree of Life' in the thicket of the phylogenetic forest
Source: J Biol. 2009 Jul 13;8(6):59. doi: 10.1186/jbiol159 (PMC2737373; doi:10.1186/jbiol159)
Supplement: Additional data file 3 — A list of the 102 COGs that are represented in at least 90 of the100 selected archaea and bacteria. [file jbiol159-S3.doc]

**The 102 Clusters of Orthologous Genes that are represented in at least 90 of the 100 selected archaea and bacteria.**

| **1) Cluster** | **2) Function** | **3) Functional category** | | **4) Sequences** | **5) Species** | **6) Separation score** | **7) Missing in:** | | **8) Archaea** | **9) Bacteria** | **10) Crenarchaeota** | **11) Euryarchaeota** | **12) Nanoarchaeota** | **13)Acidobacteria** | **14) Aquificae** | **15) Bacteroidetes** | **16) Chlamydiae** | **17) Chlorobi** | **18) Chloroflexi** | **19) Cyanobacteria** | **120) Deinococci** | **21) Firmicutes** | **22) Fusobacteria** | **23) Lentisphaerae** | **24) Planctomycetes** | **25) Proteobacteria-Alpha** | **26) Proteobacteria-Beta** | **27) Proteobacteria-Delta** | **28) Proteobacteria-Epsilon** | **29) Proteobacteria-Gamma** | **30) Spirochaetes** | **31) Thermotogae** | **32) Verrucomicrobia** |
| --- | --- | --- | --- | --- | --- | --- | --- | --- | --- | --- | --- | --- | --- | --- | --- | --- | --- | --- | --- | --- | --- | --- | --- | --- | --- | --- | --- | --- | --- | --- | --- | --- | --- |
| **COG0098** | Ribosomal protein S5 | J | Translation, ribosomal structure and biogenesis | 100 | 100 | 1.00 | 0 |  | 0 | 0 |  |  |  |  |  |  |  |  |  |  |  |  |  |  |  |  |  |  |  |  |  |  |  |
| **COG0085** | DNA-directed RNA polymerase, beta subunit/140 kD subunit | K | Transcription | 169 | 99 | 1.00 | 1 | Burma01Bp | 0 | 1 |  |  |  |  |  |  |  |  |  |  |  |  |  |  |  |  | 1 |  |  |  |  |  |  |
| **COG0086** | DNA-directed RNA polymerase, beta' subunit/160 kD subunit | K | Transcription | 160 | 100 | 1.00 | 0 |  | 0 | 0 |  |  |  |  |  |  |  |  |  |  |  |  |  |  |  |  |  |  |  |  |  |  |  |
| **COG0541** | Signal recognition particle GTPase | U | Intracellular trafficking, secretion, and vesicular transport | 98 | 97 | 1.00 | 3 | Naneq Dehsp01Bh Lepin01Bs | 1 | 2 |  |  | 1 |  |  |  |  |  | 1 |  |  |  |  |  |  |  |  |  |  |  | 1 |  |  |
| **COG0750** | Predicted membrane-associated Zn-dependent proteases 1 | M | Cell wall/membrane/envelope biogenesis | 180 | 95 | 1.00 | 5 | Theac Thevo Naneq Biflo01Ba Vicva01Bv | 3 | 2 |  | 2 | 1 | 1 |  |  |  |  |  |  |  |  |  | 1 |  |  |  |  |  |  |  |  |  |
| **COG0090** | Ribosomal protein L2 | J | Translation, ribosomal structure and biogenesis | 100 | 100 | 1.00 | 0 |  | 0 | 0 |  |  |  |  |  |  |  |  |  |  |  |  |  |  |  |  |  |  |  |  |  |  |  |
| **COG0093** | Ribosomal protein L14 | J | Translation, ribosomal structure and biogenesis | 100 | 100 | 1.00 | 0 |  | 0 | 0 |  |  |  |  |  |  |  |  |  |  |  |  |  |  |  |  |  |  |  |  |  |  |  |
| **COG0052** | Ribosomal protein S2 | J | Translation, ribosomal structure and biogenesis | 101 | 100 | 1.00 | 0 |  | 0 | 0 |  |  |  |  |  |  |  |  |  |  |  |  |  |  |  |  |  |  |  |  |  |  |  |
| **COG0201** | Preprotein translocase subunit SecY | U | Intracellular trafficking, secretion, and vesicular transport | 101 | 100 | 1.00 | 0 |  | 0 | 0 |  |  |  |  |  |  |  |  |  |  |  |  |  |  |  |  |  |  |  |  |  |  |  |
| **COG0195** | Transcription elongation factor | K | Transcription | 98 | 94 | 1.00 | 6 | Metja Picto Theac Thevo Naneq Opiba01Bv | 5 | 1 |  | 4 | 1 |  |  |  |  |  |  |  |  |  |  |  |  |  |  |  |  |  |  |  | 1 |
| **COG0197** | Ribosomal protein L16/L10E | J | Translation, ribosomal structure and biogenesis | 100 | 100 | 1.00 | 0 |  | 0 | 0 |  |  |  |  |  |  |  |  |  |  |  |  |  |  |  |  |  |  |  |  |  |  |  |
| **COG0532** | Translation initiation factor 2 (IF-2; GTPase) | J | Translation, ribosomal structure and biogenesis | 101 | 99 | 1.00 | 1 | Opiba01Bv | 0 | 1 |  |  |  |  |  |  |  |  |  |  |  |  |  |  |  |  |  |  |  |  |  |  | 1 |
| **COG0088** | Ribosomal protein L4 | J | Translation, ribosomal structure and biogenesis | 100 | 100 | 1.00 | 0 |  | 0 | 0 |  |  |  |  |  |  |  |  |  |  |  |  |  |  |  |  |  |  |  |  |  |  |  |
| **COG0480** | Translation elongation factors (GTPases) | J | Translation, ribosomal structure and biogenesis | 153 | 100 | 1.00 | 0 |  | 0 | 0 |  |  |  |  |  |  |  |  |  |  |  |  |  |  |  |  |  |  |  |  |  |  |  |
| **COG0051** | Ribosomal protein S10 | J | Translation, ribosomal structure and biogenesis | 104 | 100 | 1.00 | 0 |  | 0 | 0 |  |  |  |  |  |  |  |  |  |  |  |  |  |  |  |  |  |  |  |  |  |  |  |
| **COG0049** | Ribosomal protein S7 | J | Translation, ribosomal structure and biogenesis | 101 | 100 | 1.00 | 0 |  | 0 | 0 |  |  |  |  |  |  |  |  |  |  |  |  |  |  |  |  |  |  |  |  |  |  |  |
| **COG0030** | Dimethyladenosine transferase (rRNA methylation) | J | Translation, ribosomal structure and biogenesis | 104 | 98 | 0.94 | 2 | Naneq Vicva01Bv | 1 | 1 |  |  | 1 |  |  |  |  |  |  |  |  |  |  | 1 |  |  |  |  |  |  |  |  |  |
| **COG0552** | Signal recognition particle GTPase | U | Intracellular trafficking, secretion, and vesicular transport | 97 | 97 | 1.00 | 3 | Naneq Dehsp01Bh Lepin01Bs | 1 | 2 |  |  | 1 |  |  |  |  |  | 1 |  |  |  |  |  |  |  |  |  |  |  | 1 |  |  |
| **COG2812** | DNA polymerase III, gamma/tau subunits | L | Replication, recombination and repair | 132 | 97 | 1.00 | 3 | Hypbu Metbu Vicva01Bv | 2 | 1 | 1 | 1 |  |  |  |  |  |  |  |  |  |  |  | 1 |  |  |  |  |  |  |  |  |  |
| **COG0087** | Ribosomal protein L3 | J | Translation, ribosomal structure and biogenesis | 101 | 100 | 1.00 | 0 |  | 0 | 0 |  |  |  |  |  |  |  |  |  |  |  |  |  |  |  |  |  |  |  |  |  |  |  |
| **COG0343** | Queuine/archaeosine tRNA-ribosyltransferase | J | Translation, ribosomal structure and biogenesis | 101 | 96 | 0.98 | 4 | Halwa Myctu01Ba Mesfl01Bf Trepa01Bs | 1 | 3 |  | 1 |  | 1 |  |  |  |  |  |  |  | 1 |  |  |  |  |  |  |  |  | 1 |  |  |
| **COG0250** | Transcription antiterminator | K | Transcription | 108 | 100 | 1.00 | 0 |  | 0 | 0 |  |  |  |  |  |  |  |  |  |  |  |  |  |  |  |  |  |  |  |  |  |  |  |
| **COG0092** | Ribosomal protein S3 | J | Translation, ribosomal structure and biogenesis | 99 | 99 | 1.00 | 1 | Vicva01Bv | 0 | 1 |  |  |  |  |  |  |  |  |  |  |  |  |  | 1 |  |  |  |  |  |  |  |  |  |
| **COG0358** | DNA primase (bacterial type) | L | Replication, recombination and repair | 110 | 99 | 1.00 | 1 | MetmC | 1 | 0 |  | 1 |  |  |  |  |  |  |  |  |  |  |  |  |  |  |  |  |  |  |  |  |  |
| **COG0091** | Ribosomal protein L22 | J | Translation, ribosomal structure and biogenesis | 99 | 99 | 1.00 | 1 | Vicva01Bv | 0 | 1 |  |  |  |  |  |  |  |  |  |  |  |  |  | 1 |  |  |  |  |  |  |  |  |  |
| **COG0231** | Translation elongation factor P (EF-P)/translation initiation factor 5A (eIF-5A) | J | Translation, ribosomal structure and biogenesis | 106 | 95 | 1.00 | 5 | Censy Halsp Halwa Metst Lepin01Bs | 4 | 1 | 1 | 3 |  |  |  |  |  |  |  |  |  |  |  |  |  |  |  |  |  |  | 1 |  |  |
| **COG0096** | Ribosomal protein S8 | J | Translation, ribosomal structure and biogenesis | 100 | 100 | 1.00 | 0 |  | 0 | 0 |  |  |  |  |  |  |  |  |  |  |  |  |  |  |  |  |  |  |  |  |  |  |  |
| **COG0103** | Ribosomal protein S9 | J | Translation, ribosomal structure and biogenesis | 100 | 99 | 1.00 | 1 | Vicva01Bv | 0 | 1 |  |  |  |  |  |  |  |  |  |  |  |  |  | 1 |  |  |  |  |  |  |  |  |  |
| **COG0459** | Chaperonin GroEL (HSP60 family) | O | Posttranslational modification, protein turnover, chaperones | 188 | 99 | 0.94 | 1 | Mesfl01Bf | 0 | 1 |  |  |  |  |  |  |  |  |  |  |  | 1 |  |  |  |  |  |  |  |  |  |  |  |
| **COG0528** | Uridylate kinase | F | Nucleotide transport and metabolism | 99 | 99 | 0.95 | 1 | Naneq | 1 | 0 |  |  | 1 |  |  |  |  |  |  |  |  |  |  |  |  |  |  |  |  |  |  |  |  |
| **COG0462** | Phosphoribosylpyrophosphate synthetase | FE | Nucleotide transport and metabolism Amino acid transport and metabolism | 110 | 96 | 1.00 | 4 | Naneq Chltr01Bv Chlpn01Bv Ricpr01Bp | 1 | 3 |  |  | 1 |  |  |  | 2 |  |  |  |  |  |  |  |  | 1 |  |  |  |  |  |  |  |
| **COG0449** | Glucosamine 6-phosphate synthetase, contains amidotransferase and phosphosugar isomerase domains | M | Cell wall/membrane/envelope biogenesis | 113 | 93 | 0.88 | 7 | Arcfu Picto Theac Naneq Mesfl01Bf Ricpr01Bp Borbu01Bs | 4 | 3 |  | 3 | 1 |  |  |  |  |  |  |  |  | 1 |  |  |  | 1 |  |  |  |  | 1 |  |  |
| **COG0504** | CTP synthase (UTP-ammonia lyase) | F | Nucleotide transport and metabolism | 103 | 98 | 0.89 | 2 | Naneq Fusnu01Bu | 1 | 1 |  |  | 1 |  |  |  |  |  |  |  |  |  | 1 |  |  |  |  |  |  |  |  |  |  |
| **COG0081** | Ribosomal protein L1 | J | Translation, ribosomal structure and biogenesis | 100 | 100 | 1.00 | 0 |  | 0 | 0 |  |  |  |  |  |  |  |  |  |  |  |  |  |  |  |  |  |  |  |  |  |  |  |
| **COG0012** | Predicted GTPase, probable translation factor | J | Translation, ribosomal structure and biogenesis | 103 | 99 | 1.00 | 1 | Vicva01Bv | 0 | 1 |  |  |  |  |  |  |  |  |  |  |  |  |  | 1 |  |  |  |  |  |  |  |  |  |
| **COG0621** | 2-methylthioadenine synthetase | J | Translation, ribosomal structure and biogenesis | 168 | 95 | 1.00 | 5 | Halwa Chltr01Bv Mesfl01Bf Lacca01Bf Borbu01Bs | 1 | 4 |  | 1 |  |  |  |  | 1 |  |  |  |  | 2 |  |  |  |  |  |  |  |  | 1 |  |  |
| **COG0200** | Ribosomal protein L15 | J | Translation, ribosomal structure and biogenesis | 100 | 100 | 1.00 | 0 |  | 0 | 0 |  |  |  |  |  |  |  |  |  |  |  |  |  |  |  |  |  |  |  |  |  |  |  |
| **COG0361** | Translation initiation factor 1 (IF-1) | J | Translation, ribosomal structure and biogenesis | 120 | 99 | 1.00 | 1 | Blama01Bo | 0 | 1 |  |  |  |  |  |  |  |  |  |  |  |  |  |  | 1 |  |  |  |  |  |  |  |  |
| **COG0061** | Predicted sugar kinase | G | Carbohydrate transport and metabolism | 110 | 93 | 0.77 | 7 | Naneq Chltr01Bv Chlpn01Bv Deira01Bd Theth01Bd Vicva01Bv Opiba01Bv | 1 | 6 |  |  | 1 |  |  |  | 2 |  |  |  | 2 |  |  | 1 |  |  |  |  |  |  |  |  | 1 |
| **COG0099** | Ribosomal protein S13 | J | Translation, ribosomal structure and biogenesis | 100 | 100 | 1.00 | 0 |  | 0 | 0 |  |  |  |  |  |  |  |  |  |  |  |  |  |  |  |  |  |  |  |  |  |  |  |
| **COG0094** | Ribosomal protein L5 | J | Translation, ribosomal structure and biogenesis | 100 | 100 | 1.00 | 0 |  | 0 | 0 |  |  |  |  |  |  |  |  |  |  |  |  |  |  |  |  |  |  |  |  |  |  |  |
| **COG0215** | Cysteinyl-tRNA synthetase | J | Translation, ribosomal structure and biogenesis | 96 | 93 | 0.86 | 7 | Metth Metja Metla Metcu Metka Metbu Metsa | 7 | 0 |  | 7 |  |  |  |  |  |  |  |  |  |  |  |  |  |  |  |  |  |  |  |  |  |
| **COG0013** | Alanyl-tRNA synthetase | J | Translation, ribosomal structure and biogenesis | 126 | 98 | 1.00 | 2 | Vicva01Bv Opiba01Bv | 0 | 2 |  |  |  |  |  |  |  |  |  |  |  |  |  | 1 |  |  |  |  |  |  |  |  | 1 |
| **COG0080** | Ribosomal protein L11 | J | Translation, ribosomal structure and biogenesis | 99 | 99 | 1.00 | 1 | Vicva01Bv | 0 | 1 |  |  |  |  |  |  |  |  |  |  |  |  |  | 1 |  |  |  |  |  |  |  |  |  |
| **COG0244** | Ribosomal protein L10 | J | Translation, ribosomal structure and biogenesis | 98 | 96 | 1.00 | 4 | Hypbu Bacth01Bb Flajo01Bb Cythu01Bb | 1 | 3 | 1 |  |  |  |  | 3 |  |  |  |  |  |  |  |  |  |  |  |  |  |  |  |  |  |
| **COG0468** | RecA/RadA recombinase | L | Replication, recombination and repair | 159 | 100 | 1.00 | 0 |  | 0 | 0 |  |  |  |  |  |  |  |  |  |  |  |  |  |  |  |  |  |  |  |  |  |  |  |
| **COG0100** | Ribosomal protein S11 | J | Translation, ribosomal structure and biogenesis | 100 | 100 | 1.00 | 0 |  | 0 | 0 |  |  |  |  |  |  |  |  |  |  |  |  |  |  |  |  |  |  |  |  |  |  |  |
| **COG0519** | GMP synthase, PP-ATPase domain/subunit | F | Nucleotide transport and metabolism | 127 | 92 | 0.98 | 8 | Hypbu Stama Thepe Naneq CanPr01Bv Chltr01Bv Ricpr01Bp Trepa01Bs | 4 | 4 | 3 |  | 1 |  |  |  | 2 |  |  |  |  |  |  |  |  | 1 |  |  |  |  | 1 |  |  |
| **COG0329** | Dihydrodipicolinate synthase/N-acetylneuraminate lyase | EM | Amino acid transport and metabolism Cell wall/membrane/envelope biogenesis | 189 | 92 | 0.75 | 8 | Censy Hypbu Stama Naneq Deira01Bd Mesfl01Bf Blama01Bo Agrtu01Bp Borbu01Bs Trepa01Bs | 4 | 6 | 3 |  | 1 |  |  |  |  |  |  |  | 1 | 1 |  |  | 1 | 1 |  |  |  |  | 2 |  |  |
| **COG0130** | Pseudouridine synthase | J | Translation, ribosomal structure and biogenesis | 105 | 99 | 1.00 | 1 | Helpy01Bp | 0 | 1 |  |  |  |  |  |  |  |  |  |  |  |  |  |  |  |  |  |  | 1 |  |  |  |  |
| **COG0102** | Ribosomal protein L13 | J | Translation, ribosomal structure and biogenesis | 99 | 99 | 1.00 | 1 | Vicva01Bv | 0 | 1 |  |  |  |  |  |  |  |  |  |  |  |  |  | 1 |  |  |  |  |  |  |  |  |  |
| **COG0097** | Ribosomal protein L6P/L9E | J | Translation, ribosomal structure and biogenesis | 100 | 100 | 1.00 | 0 |  | 0 | 0 |  |  |  |  |  |  |  |  |  |  |  |  |  |  |  |  |  |  |  |  |  |  |  |
| **COG0525** | Valyl-tRNA synthetase | J | Translation, ribosomal structure and biogenesis | 105 | 100 | 0.93 | 0 |  | 0 | 0 |  |  |  |  |  |  |  |  |  |  |  |  |  |  |  |  |  |  |  |  |  |  |  |
| **COG0185** | Ribosomal protein S19 | J | Translation, ribosomal structure and biogenesis | 100 | 100 | 1.00 | 0 |  | 0 | 0 |  |  |  |  |  |  |  |  |  |  |  |  |  |  |  |  |  |  |  |  |  |  |  |
| **COG0127** | Xanthosine triphosphate pyrophosphatase | F | Nucleotide transport and metabolism | 98 | 96 | 1.00 | 4 | Naneq Ricpr01Bp Helpy01Bp Opiba01Bv | 1 | 3 |  |  | 1 |  |  |  |  |  |  |  |  |  |  |  |  | 1 |  |  | 1 |  |  |  | 1 |
| **COG0148** | Enolase | G | Carbohydrate transport and metabolism | 110 | 96 | 0.97 | 4 | Naneq Vicva01Bv Ricpr01Bp Opiba01Bv | 1 | 3 |  |  | 1 |  |  |  |  |  |  |  |  |  |  | 1 |  | 1 |  |  |  |  |  |  | 1 |
| **COG1109** | Phosphomannomutase | G | Carbohydrate transport and metabolism | 223 | 99 | 0.68 | 1 | Naneq | 1 | 0 |  |  | 1 |  |  |  |  |  |  |  |  |  |  |  |  |  |  |  |  |  |  |  |  |
| **COG0112** | Glycine/serine hydroxymethyltransferase | E | Amino acid transport and metabolism | 107 | 95 | 0.83 | 5 | Thepe MetmC Metmp Naneq Fusnu01Bu | 4 | 1 | 1 | 2 | 1 |  |  |  |  |  |  |  |  |  | 1 |  |  |  |  |  |  |  |  |  |  |
| **COG0172** | Seryl-tRNA synthetase | J | Translation, ribosomal structure and biogenesis | 100 | 98 | 0.83 | 2 | Metbu Metsa | 2 | 0 |  | 2 |  |  |  |  |  |  |  |  |  |  |  |  |  |  |  |  |  |  |  |  |  |
| **COG0143** | Methionyl-tRNA synthetase | J | Translation, ribosomal structure and biogenesis | 102 | 100 | 0.74 | 0 |  | 0 | 0 |  |  |  |  |  |  |  |  |  |  |  |  |  |  |  |  |  |  |  |  |  |  |  |
| **COG0048** | Ribosomal protein S12 | J | Translation, ribosomal structure and biogenesis | 102 | 100 | 1.00 | 0 |  | 0 | 0 |  |  |  |  |  |  |  |  |  |  |  |  |  |  |  |  |  |  |  |  |  |  |  |
| **COG0186** | Ribosomal protein S17 | J | Translation, ribosomal structure and biogenesis | 102 | 100 | 1.00 | 0 |  | 0 | 0 |  |  |  |  |  |  |  |  |  |  |  |  |  |  |  |  |  |  |  |  |  |  |  |
| **COG0016** | Phenylalanyl-tRNA synthetase alpha subunit | J | Translation, ribosomal structure and biogenesis | 99 | 99 | 0.95 | 1 | Opiba01Bv | 0 | 1 |  |  |  |  |  |  |  |  |  |  |  |  |  |  |  |  |  |  |  |  |  |  | 1 |
| **COG0089** | Ribosomal protein L23 | J | Translation, ribosomal structure and biogenesis | 99 | 99 | 1.00 | 1 | Naneq | 1 | 0 |  |  | 1 |  |  |  |  |  |  |  |  |  |  |  |  |  |  |  |  |  |  |  |  |
| **COG0126** | 3-phosphoglycerate kinase | G | Carbohydrate transport and metabolism | 103 | 98 | 0.98 | 2 | Naneq Ricpr01Bp | 1 | 1 |  |  | 1 |  |  |  |  |  |  |  |  |  |  |  |  | 1 |  |  |  |  |  |  |  |
| **COG0149** | Triosephosphate isomerase | G | Carbohydrate transport and metabolism | 95 | 95 | 1.00 | 5 | Aerpe Halma Naneq Vicva01Bv Ricpr01Bp | 3 | 2 | 1 | 1 | 1 |  |  |  |  |  |  |  |  |  |  | 1 |  | 1 |  |  |  |  |  |  |  |
| **COG0452** | Phosphopantothenoylcysteine synthetase/decarboxylase | H | Coenzyme transport and metabolism | 105 | 91 | 1.00 | 9 | Thepe Naneq Biflo01Ba CanPr01Bv Chltr01Bv Chlpn01Bv Mesfl01Bf Ricpr01Bp Trepa01Bs | 2 | 7 | 1 |  | 1 | 1 |  |  | 3 |  |  |  |  | 1 |  |  |  | 1 |  |  |  |  | 1 |  |  |
| **COG0060** | Isoleucyl-tRNA synthetase | J | Translation, ribosomal structure and biogenesis | 104 | 100 | 0.78 | 0 |  | 0 | 0 |  |  |  |  |  |  |  |  |  |  |  |  |  |  |  |  |  |  |  |  |  |  |  |
| **COG0492** | Thioredoxin reductase | O | Posttranslational modification, protein turnover, chaperones | 197 | 96 | 0.80 | 4 | Hypbu Metla Metcu Methu | 4 | 0 | 1 | 3 |  |  |  |  |  |  |  |  |  |  |  |  |  |  |  |  |  |  |  |  |  |
| **COG0018** | Arginyl-tRNA synthetase | J | Translation, ribosomal structure and biogenesis | 103 | 100 | 0.76 | 0 |  | 0 | 0 |  |  |  |  |  |  |  |  |  |  |  |  |  |  |  |  |  |  |  |  |  |  |  |
| **COG0455** | ATPases involved in chromosome partitioning | D | Cell cycle control, cell division, chromosome partitioning | 206 | 95 | 0.68 | 5 | Chltr01Bv Chlpn01Bv Mesfl01Bf Lacca01Bf Opiba01Bv | 0 | 5 |  |  |  |  |  |  | 2 |  |  |  |  | 2 |  |  |  |  |  |  |  |  |  |  | 1 |
| **COG0008** | Glutamyl- and glutaminyl-tRNA synthetases | J | Translation, ribosomal structure and biogenesis | 147 | 100 | 0.97 | 0 |  | 0 | 0 |  |  |  |  |  |  |  |  |  |  |  |  |  |  |  |  |  |  |  |  |  |  |  |
| **COG2890** | Methylase of polypeptide chain release factors | J | Translation, ribosomal structure and biogenesis | 123 | 95 | 1.00 | 5 | Censy Aerpe Sulto Arcfu Metka | 5 | 0 | 3 | 2 |  |  |  |  |  |  |  |  |  |  |  |  |  |  |  |  |  |  |  |  |  |
| **COG0256** | Ribosomal protein L18 | J | Translation, ribosomal structure and biogenesis | 100 | 100 | 1.00 | 0 |  | 0 | 0 |  |  |  |  |  |  |  |  |  |  |  |  |  |  |  |  |  |  |  |  |  |  |  |
| **COG0540** | Aspartate carbamoyltransferase, catalytic chain | F | Nucleotide transport and metabolism | 96 | 92 | 0.82 | 8 | Naneq CanPr01Bv Chltr01Bv Chlpn01Bv Mesfl01Bf Ricpr01Bp Borbu01Bs Trepa01Bs | 1 | 7 |  |  | 1 |  |  |  | 3 |  |  |  |  | 1 |  |  |  | 1 |  |  |  |  | 2 |  |  |
| **COG0167** | Dihydroorotate dehydrogenase | F | Nucleotide transport and metabolism | 124 | 92 | 0.85 | 8 | Naneq CanPr01Bv Chltr01Bv Chlpn01Bv Mesfl01Bf Ricpr01Bp Borbu01Bs Trepa01Bs | 1 | 7 |  |  | 1 |  |  |  | 3 |  |  |  |  | 1 |  |  |  | 1 |  |  |  |  | 2 |  |  |
| **COG0164** | Ribonuclease HII | L | Replication, recombination and repair | 95 | 94 | 1.00 | 6 | Vicva01Bv Blama01Bo Plama01Bo Rhoba01Bo Trepa01Bs Opiba01Bv | 0 | 6 |  |  |  |  |  |  |  |  |  |  |  |  |  | 1 | 3 |  |  |  |  |  | 1 |  | 1 |
| **COG0495** | Leucyl-tRNA synthetase | J | Translation, ribosomal structure and biogenesis | 111 | 99 | 0.94 | 1 | Opiba01Bv | 0 | 1 |  |  |  |  |  |  |  |  |  |  |  |  |  |  |  |  |  |  |  |  |  |  | 1 |
| **COG0020** | Undecaprenyl pyrophosphate synthase | I | Lipid transport and metabolism | 123 | 98 | 0.95 | 2 | Naneq Mesfl01Bf | 1 | 1 |  |  | 1 |  |  |  |  |  |  |  |  | 1 |  |  |  |  |  |  |  |  |  |  |  |
| **COG0461** | Orotate phosphoribosyltransferase | F | Nucleotide transport and metabolism | 98 | 93 | 0.66 | 7 | Naneq CanPr01Bv Chltr01Bv Mesfl01Bf Ricpr01Bp Borbu01Bs Trepa01Bs | 1 | 6 |  |  | 1 |  |  |  | 2 |  |  |  |  | 1 |  |  |  | 1 |  |  |  |  | 2 |  |  |
| **COG0284** | Orotidine-5'-phosphate decarboxylase | F | Nucleotide transport and metabolism | 97 | 91 | 0.69 | 9 | Naneq CanPr01Bv Chltr01Bv Chlpn01Bv Mesfl01Bf Ricpr01Bp Borbu01Bs Lepin01Bs Trepa01Bs | 1 | 8 |  |  | 1 |  |  |  | 3 |  |  |  |  | 1 |  |  |  | 1 |  |  |  |  | 3 |  |  |
| **COG0522** | Ribosomal protein S4 and related proteins | J | Translation, ribosomal structure and biogenesis | 105 | 100 | 1.00 | 0 |  | 0 | 0 |  |  |  |  |  |  |  |  |  |  |  |  |  |  |  |  |  |  |  |  |  |  |  |
| **COG0024** | Methionine aminopeptidase | J | Translation, ribosomal structure and biogenesis | 123 | 99 | 1.00 | 1 | Cloac01Bf | 0 | 1 |  |  |  |  |  |  |  |  |  |  |  | 1 |  |  |  |  |  |  |  |  |  |  |  |
| **COG0142** | Geranylgeranyl pyrophosphate synthase | H | Coenzyme transport and metabolism | 191 | 97 | 0.68 | 3 | Naneq Mesfl01Bf Opiba01Bv | 1 | 2 |  |  | 1 |  |  |  |  |  |  |  |  | 1 |  |  |  |  |  |  |  |  |  |  | 1 |
| **COG0441** | Threonyl-tRNA synthetase | J | Translation, ribosomal structure and biogenesis | 146 | 99 | 0.87 | 1 | Vicva01Bv | 0 | 1 |  |  |  |  |  |  |  |  |  |  |  |  |  | 1 |  |  |  |  |  |  |  |  |  |
| **COG0124** | Histidyl-tRNA synthetase | J | Translation, ribosomal structure and biogenesis | 103 | 100 | 0.74 | 0 |  | 0 | 0 |  |  |  |  |  |  |  |  |  |  |  |  |  |  |  |  |  |  |  |  |  |  |  |
| **COG0136** | Aspartate-semialdehyde dehydrogenase | E | Amino acid transport and metabolism | 103 | 91 | 0.89 | 9 | Hypbu Stama Thepe Thevo Naneq Mesfl01Bf Fusnu01Bu Borbu01Bs Trepa01Bs | 5 | 4 | 3 | 1 | 1 |  |  |  |  |  |  |  |  | 1 | 1 |  |  |  |  |  |  |  | 2 |  |  |
| **COG0198** | Ribosomal protein L24 | J | Translation, ribosomal structure and biogenesis | 96 | 96 | 1.00 | 4 | Censy Sulto Fusnu01Bu Metpe01Bp | 2 | 2 | 2 |  |  |  |  |  |  |  |  |  |  |  | 1 |  |  |  | 1 |  |  |  |  |  |  |
| **COG0527** | Aspartokinases | E | Amino acid transport and metabolism | 132 | 91 | 0.72 | 9 | Stama Thepe Thevo Naneq Mesfl01Bf Fusnu01Bu Borbu01Bs Trepa01Bs Opiba01Bv | 4 | 5 | 2 | 1 | 1 |  |  |  |  |  |  |  |  | 1 | 1 |  |  |  |  |  |  |  | 2 |  | 1 |
| **COG0071** | Molecular chaperone (small heat shock protein) | O | Posttranslational modification, protein turnover, chaperones | 216 | 91 | 0.87 | 9 | Chltr01Bv Chlpn01Bv Proma01Bc Mesfl01Bf Fusnu01Bu Neime01Bp Helpy01Bp Borbu01Bs Trepa01Bs | 0 | 9 |  |  |  |  |  |  | 2 |  |  | 1 |  | 1 | 1 |  |  |  | 1 |  | 1 |  | 2 |  |  |
| **COG0177** | Predicted EndoIII-related endonuclease | L | Replication, recombination and repair | 127 | 99 | 0.74 | 1 | Mesfl01Bf | 0 | 1 |  |  |  |  |  |  |  |  |  |  |  | 1 |  |  |  |  |  |  |  |  |  |  |  |
| **COG0237** | Dephospho-CoA kinase | H | Coenzyme transport and metabolism | 105 | 99 | 1.00 | 1 | Naneq | 1 | 0 |  |  | 1 |  |  |  |  |  |  |  |  |  |  |  |  |  |  |  |  |  |  |  |  |
| **COG0105** | Nucleoside diphosphate kinase | F | Nucleotide transport and metabolism | 96 | 95 | 0.68 | 5 | Thepe Biflo01Ba Mesfl01Bf Fusnu01Bu Thema01Bt | 1 | 4 | 1 |  |  | 1 |  |  |  |  |  |  |  | 1 | 1 |  |  |  |  |  |  |  |  | 1 |  |
| **COG1080** | Phosphoenolpyruvate-protein kinase (PTS system EI component in bacteria) | G | Carbohydrate transport and metabolism | 154 | 94 | 0.74 | 6 | Hypbu Naneq Myctu01Ba Flajo01Bb Cythu01Bb Proma01Bc | 2 | 4 | 1 |  | 1 | 1 |  | 2 |  |  |  | 1 |  |  |  |  |  |  |  |  |  |  |  |  |  |
| **COG0180** | Tryptophanyl-tRNA synthetase | J | Translation, ribosomal structure and biogenesis | 159 | 100 | 1.00 | 0 |  | 0 | 0 |  |  |  |  |  |  |  |  |  |  |  |  |  |  |  |  |  |  |  |  |  |  |  |
| **COG0057** | Glyceraldehyde-3-phosphate dehydrogenase/erythrose-4-phosphate dehydrogenase | G | Carbohydrate transport and metabolism | 132 | 97 | 0.95 | 3 | Metsa Naneq Ricpr01Bp | 2 | 1 |  | 1 | 1 |  |  |  |  |  |  |  |  |  |  |  |  | 1 |  |  |  |  |  |  |  |
| **COG0125** | Thymidylate kinase | F | Nucleotide transport and metabolism | 115 | 96 | 0.82 | 4 | Naneq Bacth01Bb Flajo01Bb Cloac01Bf | 1 | 3 |  |  | 1 |  |  | 2 |  |  |  |  |  | 1 |  |  |  |  |  |  |  |  |  |  |  |
| **COG0037** | Predicted ATPase of the PP-loop superfamily implicated in cell cycle control | D | Cell cycle control, cell division, chromosome partitioning | 170 | 99 | 0.89 | 1 | Halwa | 1 | 0 |  | 1 |  |  |  |  |  |  |  |  |  |  |  |  |  |  |  |  |  |  |  |  |  |
| **COG0006** | Xaa-Pro aminopeptidase | E | Amino acid transport and metabolism | 180 | 98 | 0.63 | 2 | Naneq Vicva01Bv | 1 | 1 |  |  | 1 |  |  |  |  |  |  |  |  |  |  | 1 |  |  |  |  |  |  |  |  |  |
| **COG0009** | Putative translation factor (SUA5) | J | Translation, ribosomal structure and biogenesis | 126 | 97 | 0.66 | 3 | Naneq Helpy01Bp Sulsp02Bp | 1 | 2 |  |  | 1 |  |  |  |  |  |  |  |  |  |  |  |  |  |  |  | 2 |  |  |  |  |
| **COG0171** | NAD synthase | H | Coenzyme transport and metabolism | 112 | 94 | 0.76 | 6 | Naneq CanPr01Bv Chltr01Bv Chlpn01Bv Ricpr01Bp Opiba01Bv | 1 | 5 |  |  | 1 |  |  |  | 3 |  |  |  |  |  |  |  |  | 1 |  |  |  |  |  |  | 1 |
| **COG0537** | Diadenosine tetraphosphate (Ap4A) hydrolase and other HIT family hydrolases | FGR | Nucleotide transport and metabolism Carbohydrate transport and metabolism General function prediction only | 167 | 94 | 0.67 | 6 | Uncme Metst Methu Metka Trepa01Bs Thema01Bt | 4 | 2 |  | 4 |  |  |  |  |  |  |  |  |  |  |  |  |  |  |  |  |  |  | 1 | 1 |  |

The columns: 1) COGs number; 2) Specific function; 3) Functional category; 4) Number of sequences; 5) Number of species; 6) Separation score SS_B/A_; 7) Species where the COG is missing; 8) Number of archaeal species missing each COG; 9) Number of bacterial species missing each COG; 10-32). Missing COGs are classified by taxa: for each taxon, the number of species in which the given COG is missing is indicated.
